# Selected Papers in 2023–2024 in the ‘Polymer Applications’ Section

**DOI:** 10.3390/polym17243245

**Published:** 2025-12-05

**Authors:** Hyeonseok Yoon

**Affiliations:** 1 School of Polymer Science and Engineering, Chonnam National University, 77 Yongbong-ro, Buk-gu, Gwangju 61186, Republic of Korea; hyoon@chonnam.ac.kr; 2 Department of Polymer Engineering, Graduate School, Chonnam National University, 77 Yongbong-ro, Buk-gu, Gwangju 61186, Republic of Korea

During 2023 and 2024, the Section “Polymer Applications” of the MDPI journal *Polymers* achieved an exceptional level of academic productivity, as evidenced by the publication of 995 peer-reviewed articles and several high-impact Special Issues. These works encompassed a diverse range of relevant topics, such as the functional integration of bio-plastic packaging materials, the enhancement of thermal stability in 3D-printed polymers, and the development of uniform polymeric systems for high-performance hydrogels. Furthermore, numerous studies advanced the understanding of energy storage efficiency adsorptive functionality, and the design of multifunctional polymer architectures tailored for emerging technologies. Complementing these experimental efforts, computational modeling and simulation approaches were extensively employed to predict, analyze, and optimize polymer behaviors across various application domains.

In the food packaging industry, Federico Barrino et al. developed fully degradable biomaterials based on polybutylene succinate, enriched with virgin olive oil and coconut oil and optimized at a concentration of 3 wt%. These films exhibited stable mechanical properties, high hydrophobicity, and particularly reduced oxidation and mold and bacteria growth on fresh fruit slices. Accordingly, food storage tests on kiwi and apple slices showed the ability to reduce oxidative browning. In particular, the presence of virgin olive oil in the film significantly reduced the growth of fungi and bacteria [1].

For polymer composites in biomedical applications, a novel aloe vera-based hydrogel was developed by Mariana Chelu, Adina Magdalena Musuc, and colleagues using allantoin, xanthan gum, and salicylic acid, with aloe vera concentrations ranging from 5 to 20% (*w*/*v*). Comprehensive characterization confirmed homogeneous morphology, good mechanical integrity, and favorable physicochemical properties. Among the tested formulations, the hydrogel containing 10% aloe vera showed the highest tensile strength, elasticity, and absorption capacity, as well as excellent spreadability. These features highlight aloe vera-based hydrogels as the optimal composition for biomedical and wound-healing applications, owing to its balanced structural stability and enhanced bioactivity [2].

In addition, Enkhtuya Majigsuren, Ganchimeg Yunden, and co-workers developed particle composites from biopolymer chitosan and natural clay from Tsogt-Ovoo mine, Mongolia, for heavy metal adsorption from aqueous solutions, aiming to improve the properties (porosity, thermal stability, and density) of pure chitosan particles. Natural clay was pretreated with acid and heat to remove impurities and then mixed with chitosan in different ratios to form composite particles. The adsorption process of Cr(III) and Cr(VI) was investigated according to solution pH, time, temperature, initial concentration, and mass of composite particles. The results showed that chitosan/clay composite particles with ratios of 8:1 and 8:2 had the highest adsorption capacity (23.5 and 17.31 mg·g^−1^) for Cr(III) and Cr(VI) at the optimal conditions of pH = 4 and pH = 3, respectively. Chromium ions were adsorbed in the initial form of Cr(III) and Cr(VI) without undergoing an oxidation–reduction reaction, with Cr(III) bound to the hydroxyl group and Cr(VI) bound to the amino group of the composite particles. Kinetic, thermodynamic, and isotherm analyses showed that the interaction between the composite particles and Cr(III) and Cr(VI) ions was a second-order endothermic reaction, consistent with the Langmuir isotherm model. The study concluded that the composite particles could be used as an effective adsorbent for the removal of chromium ions [3].

In the field of functional polymer materials, Uijung Hwang and Hyun Wook Jung et al. developed a pH-responsive poly(ethylene glycol) (PEG)/poly(acrylic acid) (PAA) interpenetrating polymer network hydrogel system through a two-step UV process: first, PEG hydrogels were formed by free-radical polymerization, then immersed in solutions of acrylic acid monomer and poly(ethylene glycol) dimethacrylate linker with different molar ratios, and then UV irradiated for the second time to form independent PAA networks inside the PEG network. The study showed that the PAA network exhibits pH-sensitive properties: below the pKa (4.3) of acrylic acid, hydrogen bonding between PEG and PAA chains causes hydrogel contraction; above the pKa, PAA ionization creates electrostatic repulsion and osmotic pressure, which increases water absorption and modulus due to the limited stretchability of the PEG network chains. Adjusting the PAA network bonding density allows for precise tuning of hydrogel properties [4].

In the dietary supplements sector, a study by Aleksandra Vojvodić Cebin and Draženka Komes developed gelatin gummies containing mountain germander extract—a source of phenylethanoid glycosides (PhEG)—and prebiotics, aiming to provide a functional product for health-conscious consumers. Sucrose and glucose syrup were partially or completely replaced by xylitol, maltitol and prebiotic poly/oligosaccharides. Physicochemical, textural, and sensory parameters were evaluated after production and 2 months of storage. The gummies containing fructooligosaccharides (FOS) and xylooligosaccharides (XOS) maintained their characteristic shape at all three sugar levels, while the samples containing inulin and without prebiotics were contaminated with mold or had changes in shape/structure. Candy color significantly darkened over time, especially for the XOS sample, but remained consistent with that of a herbal product. PhEG was highly stable in manufacturing (~90%) and storage, with echinacoside being the dominant component. Candy texture was elastic with high positive correlations between resilience, cohesiveness, springiness, and chewiness; however, inulin had the greatest influence on elasticity. Sensory evaluation showed high transparency, moderate sweetness, and bitterness, with a slight reduction in bitterness during storage increasing overall acceptability. FOS and XOS samples had the highest potential for success, providing 32–40 mg PhEG and up to 4.5 g fiber per serving, with XOS having the advantage of a lower effective dose and sustainable provenance with a suggested storage life of 1.5 months due to its high relative humidity content [5].

With respect to the application possibilities of polymers in 3D printing technology, Mariam Shbanah, Tünde Anna Kovács, and their co-workers evaluated the influence of heat treatment on the mechanical and structural properties of PLA produced by 3D printing technology. Standard tensile specimens were printed in the vertical and horizontal orientations, heat-treated at 55 °C, 65 °C, and 80 °C for 5 h, and then kept at 20 °C for 15 h. The tensile test results confirmed the dependence of mechanical properties on the printing direction, with the vertical printed specimen achieving the highest increase in ultimate tensile strength after heat treatment at 65 °C, while all heat treatment modes improved the ultimate tensile strength of the horizontal printed specimen. Microscopic analysis of the fracture surface and structure of the samples after heat treatment at 65 °C showed a significant decrease in porosity, while the sample dimensions did not change significantly at different temperatures. The increase in tensile strength can be explained by improved crystallinity and reduced residual stresses in the printed layers as each layer experienced different temperatures and times during printing. The study concluded that heat treatment is a suitable method to increase the mechanical properties of PLA after 3D printing, with print orientation still being the most important factor affecting tensile strength, but this can be significantly improved through appropriate heat treatment [6].

To address the design and verification requirements of components under real operating conditions, as 3D printing evolves from aesthetic purposes to small-batch and pre-series production, Francesco Bandinelli, Alberto Morena et al. developed an elasto-plastic mechanical modeling strategy for short fiber-reinforced polymer materials produced by 3D printing technology. Due to strong anisotropy, the model is based on a transversely isotropic hypothesis with an elasticity matrix for the elastic domain and a combination of Hill’s yield criterion and Voce’s hardening law for the plastic domain. Material properties were determined through tensile tests on dog bone specimens printed in multiple orientations, with five elasticity matrix parameters and four Hill’s criterion parameters. The model was calibrated using the Levi–Mises plastic flow rule and volume conservation assumption. Results showed good agreement between simulation and experiment in describing stress up to maximum load, but strain behavior requires refinement due to difficulties in evaluating equivalent deformation and the relatively porous nature of 3D-printed materials [7].

To more concretely evaluate the real-world impact of 3D-printed products, Luka Šimunović, Senka Meštrović et al. evaluated the durability and abrasion resistance of thermosetting polyurethane polymers used in clear aligners against common staining agents (coffee, black tea, Coca-Cola, and Red Bull) in terms of color and chemical stability. Thermoformed and 3D-printed polyurethane aligners from four brands were exposed to beverages to evaluate color change using the VITA Easyshade^®^ compact colorimeter after 24, 48, 72, and 7 days, along with chemical stability via attenuated total reflection-FTIR (ATR-FTIR) spectroscopy. Accordingly, ATR-FTIR analysis revealed compositional differences with different variations upon beverage exposure affecting polymer bond integrity and specifically highlighted coffee as the strongest staining agent. The polyurethane 3D-printed trays changed color more than the thermoformed trays, emphasizing the importance of manufacturing techniques and the outer cover layer in stain resistance [8].

In the field of energy applications, Changhai Zhang and Qingguo Chi et al. used heat treatment to improve the energy storage performance of PMMA/PVDF composite materials to meet the demand for clean energy and overcome the limitations of commercial biaxially oriented polypropylene capacitors. PMMA/PVDF composite films were fabricated by solution casting with different ratios with good compatibility. The effects of PMMA doping ratio and heat treatment temperature on the microstructure and material properties were systematically investigated, thereby determining the optimal ratio of PMMA:PVDF = 5.5:4.5. At a treatment temperature of 120 °C, the breakdown strength of the composite increased from 389 kV/mm to 729.42 kV/mm, resulting in an energy storage density of 21.12 J/cm^3^ and a discharge efficiency of 64.8%, which were significantly improved compared to pure PVDF. The study provides a useful method for designing polymer materials with high energy storage performance, contributing to improving the energy storage capacity of polymer matrix composites [9].

In addition, Liming Liu and Yong Zhang et al. proposed a novel strategy to enhance the piezoelectric performance of PVDF-based composites by doping DET into ceramic nanoparticles, demonstrating the potential in human–machine interaction and remote control for the Internet of Things. Accordingly, the study used di(dioctylpyrophosphate)ethylene titanate (DET) as a coupling agent to improve barium titanate (BTO) nanoparticles to overcome the challenges of poor interface compatibility between ceramic nanofillers and polymer matrix as well as low piezoelectric performance in flexible piezoelectric composites for self-powered wearable sensors. Compared with unmodified BTO, DET-BTO exhibited superior dispersibility in PVDF solution, improved interface compatibility, stress transfer efficiency, and promoted the formation of piezoelectric β-phase in PVDF. DET-BTO/PVDF nanocomposite fibers were fabricated by electrostatic spraying and formed into piezoelectric energy harvesters (PEHs), which exhibited enhanced β-phase content (~85.7%) and piezoelectric coefficient up to ~40 pC/N—the highest value among reported BTO/PVDF composites. PEHs with 3 wt% DET-BTO achieved an instantaneous power density of 276.7 nW/cm^2^ at a suitable load of 120 MΩ under a force of 18 N, 3.09 times higher than that of the unmodified sample, with good durability. Furthermore, PEHs were capable of sensing human activities with high sensitivity up to 0.817 V/N in the range of 0.05–0.1 N and were successfully applied for pulse and respiratory monitoring and voice recognition [10].

These research achievements collectively reflect the exceptional scientific endeavors undertaken in the field of *Polymers*. As we move forward into 2025, I anticipate continued progress and expansion of the Polymer Application section, supported by our dedicated Editorial Board.

We welcome your contributions on the latest developments in applications of polymers!
